# G6PD deficiency in *Plasmodium falciparum* and *Plasmodium vivax* malaria-infected Cambodian patients

**DOI:** 10.1186/1475-2875-12-171

**Published:** 2013-05-28

**Authors:** Nimol Khim, Christophe Benedet, Saorin Kim, Sim Kheng, Sovannaroth Siv, Rithea Leang, Soley Lek, Sinuon Muth, Nguon Chea, Char Meng Chuor, Socheat Duong, Alexandra Kerleguer, Pety Tor, Pheaktra Chim, Lydie Canier, Benoit Witkowski, Walter RJ Taylor, Didier Ménard

**Affiliations:** 1 Malaria Molecular Epidemiology Unit, Institut Pasteur du Cambodge, Phnom Penh, Cambodia; 2 National Center for Parasitology, Entomology and Malaria Control, Phnom Penh, Cambodia; 3 Medical Laboratory, Institut Pasteur du Cambodge, Phnom Penh, Cambodia

## Abstract

**Background:**

Glucose-6-phosphate-dehydrogenase deficiency (G6PDd) rates are unknown in malaria-infected Cambodian patients. These data are key to a rational drug policy for malaria elimination of *Plasmodium falciparum* and *Plasmodium vivax*.

**Methods:**

From September 2010–2012, a two-year survey of G6PDd and haemoglobinopathies assessed by quantitative enzyme activity assay and haemoglobin electrophoresis, respectively, was conducted in malaria-infected patients presenting to 19 health centres throughout Cambodia.

**Results:**

A total of 2,408 confirmed malaria patients of mean age 26.7 (range 2–81) years were recruited from mostly western Cambodia (n = 1,732, 71.9%); males outnumbered females by 3.9:1. *Plasmodium falciparum* was present in 1,443 (59.9%) and *P. vivax* in 965 (40.1%) patients. Mean G6PD activity was 11.6 (CI 95%: 11.4-11.8) U/g Hb, G6PDd was present in 13.9% of all patients (335/2,408) and severe G6PDd (including WHO Class I and II variants) was more common in western (158/1,732, 9.1%) *versus* eastern (21/414, 5.1%) Cambodia (*P* = 0.01). Of 997/2,408 (41.4%) had a haemoglobinopathy. Mean haemoglobin concentrations were inversely related to age: 8.1 g/dL < five years, 8.7 g/dL five to 14 years, and 10.4 g/dL >15 years (P <0.001).

**Conclusions:**

G6PDd prevalence, anaemia and haemoglobinopathies were common in malaria-infected patients. The deployment of primaquine in Cambodia should be preceded by primaquine safety studies paralleled with evaluations of easy to use tests to detect G6PDd.

## Background

Controlling malaria remains a significant global health challenge [1], especially in areas of low transmission which are seen as prime areas for malaria elimination [2]. The World Health Organization (WHO) has been urging countries for many years to use primaquine for both transmission blocking of *Plasmodium falciparum*, because it kills mature gametocytes, and as anti-relapse treatment against *Plasmodium vivax* by killing liver hypnozoites [3,4]. Primaquine is not used widely because of anxiety over its well-known propensity to cause acute haemolytic anaemia (AHA) in individuals with glucose-6-phosphate-dehydrogenase deficiency (G6PDd) [5], a scenario reported by senior Cambodian clinicians (Mey Bout Denis, Cambodian National Malaria Control Programme, CNM, personal communication), coupled with the current logistical and financial impossibility of offering G6PD screening to all malaria patients [6]. Nevertheless, the emergence of artemisinin resistance in Cambodia and other Southeast Asian countries has added considerable urgency to containing artemisinin resistant parasites by transmission blocking with primaquine [7]. Indeed, the WHO has recommended low dose primaquine (0.25 mg/kg stat) for *P. falciparum* without prior G6PD testing believing that AHA would be clinically mild [8].

Cambodia has low transmission of essentially falciparum and vivax malaria in an overall ratio of about 1:1 but there is seasonal and geographical variation. Approximately half of the population, ~3,000,000, is estimated to be at risk of malaria [9] and public sector data from 2010 show an overall incidence of 4.07 cases/1,000 population (Cambodian National Malaria Control Programme, CNM Annual Reports 2000–2009), an historically low rate but one that is higher than neighbouring countries. Controlling malaria is a high priority for Cambodia and the Royal Cambodian government has committed itself to eliminating malaria by 2025.

G6PDd is a common, X-linked hereditary enzyme deficiency affecting approximately 400 million people worldwide [10], mainly in malaria-endemic regions [11]. G6PD is a key enzyme for protecting red cells against oxidant stress by allowing the production of NADPH from the hexose monophosphate pathway. G6PDd variants number about 400 and have differing amounts of G6PD enzyme activity that are classified broadly as very severe, severe or mild [12]. Hemizygote males and homozygote females are most and least commonly affected, respectively. Heterozygote females have mixed G6PD normal and deficient red cells and their total G6PD enzyme activity and susceptibility to haemolysis depends on the balance between the expression of the normal and abnormal X chromosomes [13]. Primaquine-induced AHA is dose dependent and inversely related to G6PD enzyme activity [14]. Thus, primaquine given to individuals with mild G6PDd (e g, African A-) tends to produce mild, self-limiting AHA [15-18] but greater AHA and longer times to haemoglobin recovery in severe G6PDd (e g, Asian or B- variants) [19-22].

The G6PDd prevalence rates in Cambodia vary between 13.4 and 26.1% in males and 3.1 and 4.3% in females, depending on the sampled population (see Table 1) [20,23-27]. The severe G6PDd variant, G6PD-Viangchan, predominates in Cambodia, followed by G6PD-Mahidol, G6PD-Union, and G6PD-Coimbra [24,26,28]. The normal range (95% confidence interval (CI)) of G6PD enzyme activity in healthy Cambodians has been estimated at 11.6-12.1 U/g Hb for a population mean of 11.8 U/g Hb [24].

**Table 1:** Summary of different studies conducted in Cambodia to assess G6PD deficiency

**Authors - Journal - Date**	**Year**	**Targeted population**	**N**	**Method**	**Main findings**	**Comments**
Goueffon *et al.*, Bull. Soc. Pathol. Exot., 1969 [23]	1966	?			G6PDd = 14.5%	No abstract available
Everett *et al.*, Am. J. Trop. Med. Hyg., 1977 [20]	1975	Healthy Khmer Air Force male troops	106	?	G6PDd = 14.1% for males (100% G6PD-Mahidol) - G6PD-Mahidol enzyme activities = 4% to 11% of G6PD normal levels	Primaquine trial (15 mg/day for 2 weeks)
Monchy *et al.*, Med. Trop., 2004 [27]	2002	Children aged from 6 months to 5 years in Kandal province	151	G6PD assay activity (37°C)	G6PDd = 13.4% for males and 4.3% for females	G6PDd <2.4 U/gHb and G6PDi = 2.4-5.1 U/gHb
Louicharoen *et al.*, J. Hum. Genet., 2005 [25]	2002	Migrant Cambodian labourers in Chanthaburi province, Thailand,	108	G6PD assay activity (37°C) and PCR-RFLP	G6PDd = 26.1% for males and 3.1% for females, G6PD-ViangChan = 82.4%, G6PD-Union = 3.0%, G6PD-Coimbra = 3.0%, Unkwon = 11.6%	G6PDd <1.5 U/gHb
	2003	Cord blood samples newborns of Khmer-speaking mothers	107	G6PD assay activity (37°C)		
Matsuoka *et al.*, J. Hum. Genet., 2005 [26]	2003-2004	Battambang, Kampot, Phnom Penh and Rattanakiri provinces (13 sites)	670	G6PD Assay Kit (Dojindo Laboratories, Tokyo, Japan) and PCR-sequencing	G6PDd = 8.1% for males and 5.3% for females (in Khmer ethnic group, G6PDd = 12.6% for males and 13.8% for females; in Tum Pun group, G6PDd = 1.1% for males and in Cha Ray ethnic group, G6PDd = 3.2% for males), G6PD-ViangChan = 97.9%, G6PD-Union = 2.1%.	Divide into (1) full activity of G6PD, (2) partial deficiency group, and (3) complete deficiency group
Steenkeste *et al.*, PhD thesis, 2009	2001	8 villages and 3 ethic groups (Tum Pun, n = 698; Jaray, n = 179; Brao, n = 237)	1116	PCR-sequencing (Exons 9–12)	100% G6PD-ViangChan. G6PDd = 4.3% (3.0% for males and 5.4% for females). G6PDd = 0.3% in Tum Pun group, 2.2% in Jaray and 17.7% in Brao).	
Kim *et al.*, PlosOne, 2011 [24]	2010	4 villages in Pailin province	903	G6PD assay activity (Trinity Biotech). G6PD RDT (Access Bio) and PCR-sequencing (all exons)	G6PDd = 10.7% (15.0% for males and 6.9% for females). Fully G6PDd = 1.2%, severe G6PDd = 5.6%, mild G6PDd = 11.0%, normal G6PD = 76.6% and increased G6PD = 5.6%. G6PD-ViangChan = 95.5%, G6PD-Canton = 1.5%, G6PD-Mahidol = 1.5% and G6PD-Valladolid = 1.5%.	fully G6PDd (WHO Class I) < 0.12 U/gHb, severe G6PDd (WHO Class II): >0.12-1.2 U/gHb, mild G6PDd (WHO Class III): >1.2-7.1 U/gHb, normal G6PD (WHO Class IV): >7.1-17.7 U/gHb, increased G6PD (WHO Class V) >17.7 U/gHb

To date, there has not been an estimate of the frequencies of G6PDd in malaria-infected patients seeking anti-malarial treatment in public health facilities. Such patients would be eligible to receive primaquine for either falciparum transmission blocking or weekly antirelapse primaquine but data on weekly primaquine in Cambodian G6PDd variants are currently lacking. Obtaining G6PDd data and its association with malaria, haemoglobinopathies and anaemia would be important for the Cambodian National Malaria Control Programme (CNM) to prioritize its anti-malarial drug policy and to conduct future research on the safety of primaquine. Results of a G6PDd survey in malaria patients are reported herein.

## Methods

### Study population and site

**Figure 1:**
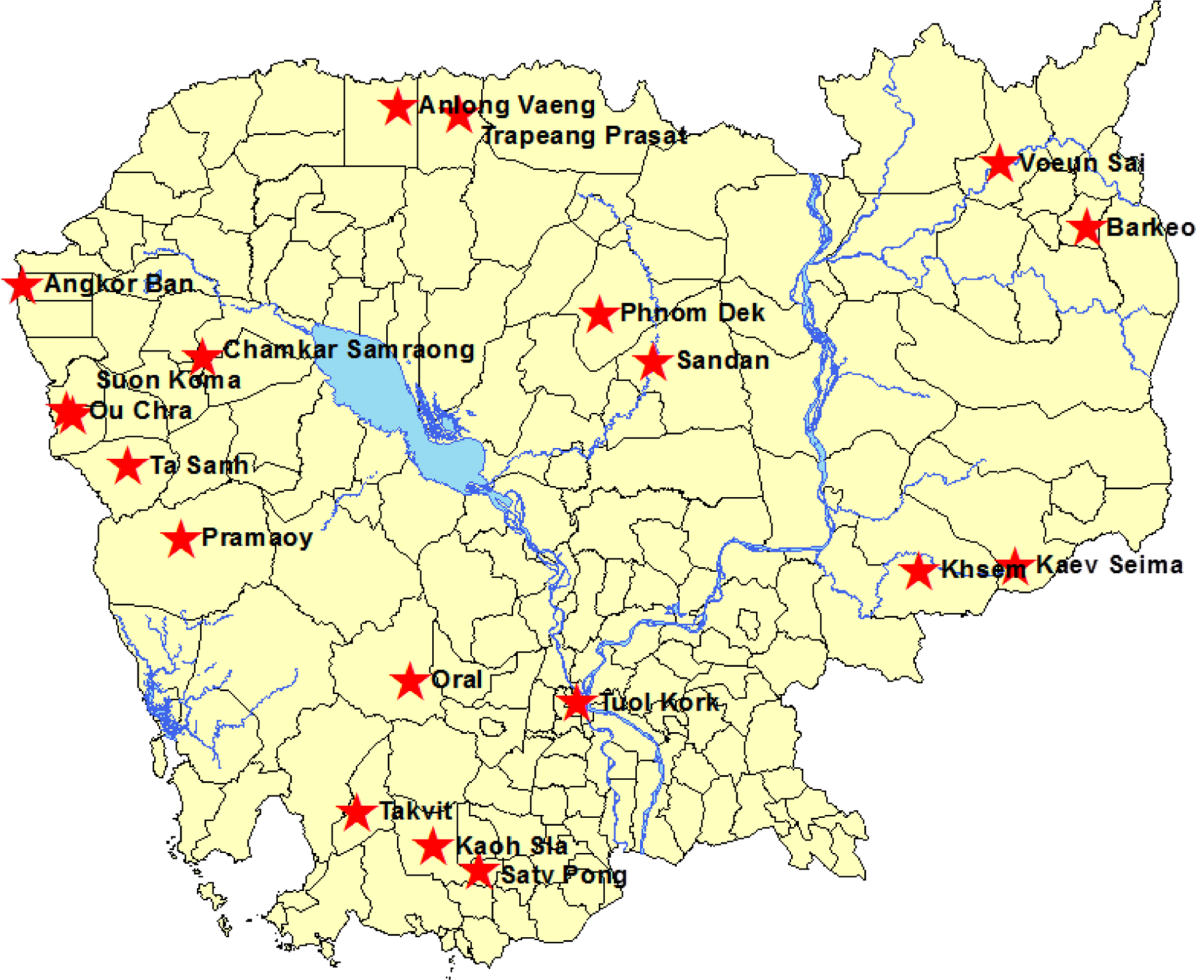
Map of Cambodia and locations of the 19 sample collection sites, Cambodia, 2010–2012.

The study took place from 2010 to 2012 at 19 public health facilities from across Cambodia (Figure 1), which are involved in the National Network for Monitoring Anti-malarial Drug Resistance in Cambodia, collaboration between CNM and Institut Pasteur du Cambodge (IPC). Malaria diagnosis was achieved in febrile patients seeking treatment, either by microscopy of Giemsa-stained malaria blood films or by a malaria rapid diagnostic test (RDT) that detects *P. falciparum* and non-*P. falciparum* parasites (CareStart™, AccessBio, USA). Malaria-positive patients or their legal guardians were asked if they would be interested to join the study. If signed informed consent was obtained, patients were allocated a study number and had blood taken. The study protocol was reviewed and approved by the Ethics Committee of the Cambodian Ministry of Health (No 160 NECHR, 28 October, 2010).

### Sample collection

Five ml of venous blood were collected into ACD-coated tubes, stored in a fridge before transport to IPC within 24 to 48 hours at +4°C in cool boxes. At IPC, repeat malaria blood films were made, stained with 3% Giemsa solution for 30 to 45 minutes, and checked for *Plasmodium* species by light microscopy. Blood samples were divided into two aliquots for (i) complete blood count (CBC), quantitative determination of G6PD activity and haemoglobin electrophoresis, and, (ii) *in vitro* anti-malarial drug sensitivity testing and detection of molecular markers related to anti-malarial drug resistance.

### Haematological parameters

The CBC was determined using a CellDyn 3200 analyzer™ (Abbott, France) after daily standardization with three different controls of all the standard parameters.

### Quantitative determination of G6PD activity

Determination of the G6PD enzyme activity was performed on the fresh blood within a maximum of 48 hours after sample collection, using the Trinity Biotech quantitative G6PD assay™ (Ref 345-UV, Trinity Biotech, USA) adapted on the Integra 400 analyzer™ (Roche Diagnostic, France), according to the manufacturer’s instructions and as described previously [24]. The reliability of the results were monitored by calibration using three different enzyme activity controls provided by Trinity Biotech (deficient level ref G5888, intermediate level ref G5029, and normal level, ref G6888) within each run. G6PD activities were expressed as units per gram of haemoglobin, U/g Hb.

G6PD deficiency was classified according to the WHO classification expressing the G6PD enzyme activity as a percentage of the population defined mean, 11.8 U/g Hb for Cambodia [24]: (i) Class I: very severely deficient, ≤1% residual activity, ≤0.12 U/g Hb, (ii) Class II: severely deficient, >1-10% residual activity, >0.12-1.2 U/g Hb, (iii) Class III: mildly deficient, >10-60% residual activity, >1.2-7.1 U/g Hb, (iv) Class IV: normal activity, >60% to 150% residual activity, >7.1-17.7 U/g Hb, and (v) Class V: increased activity, >150% residual activity, >17.7 U/g Hb.

### Capillary electrophoresis

The capillary electrophoresis was performed with the MINICAP system™ (Sebia, France) according to the manufacturer’s instructions and using controls of human blood for every run: (i) normal Hb A_2_ control (ref PN4778), (ii) normal HbA and F and abnormal HbS and C (Hb AFSC control, ref PN4792), and (iii) increased haemoglobin A_2_ fraction (ref PN4779).

Briefly, red cell haemolysate was automatically prepared on the instrument and charged molecules were separated according to their electrophoretic mobility in an alkaline buffer (pH 9.4). Relative quantification and presumptive identification of the haemoglobin fractions were performed automatically by the software. The HbA fraction was centred in the middle of the review window and resulting electrophoregrams evaluated visually for pattern abnormalities (identified in zones Z1 to Z15) to classify haemoglobinopathies as: (i) normal profile: HbA >97%, HbA_2_ =1.8-3.4%, HbF <1%, (ii) homozygous HbE: HbE > 85%, HbA = 0%, HbA_2_ = 3.4-5.3%, HbF <15%, (iii) heterozygous HbE: HbE = 25-30%, HbA = 68-73%, HbA_2_ = 2.5-4.5%, HbF < 1%, (iv) mixed heterozygous HbE and α-thalassaemia: HbA = 73-85%, HbA_2_ = 3.7-4.5%, HbF < 1%,, (v) heterozygous HbE and β-thalassaemia: HbA = 0-10%, HbA_2_ = 2.2-4.4%, HbF = 30-65%, HbE = 0-15%, and (vi) β-thalassaemia: HbA > 90%, HbA_2_ = 2.6-6.5, HbF = 3.0-7.4%, HbE = 0%.

### Data analysis

Data were entered and verified using Microsoft Excel 2010 software and analysed using MedCalc software (version 9.1.0.1, Mariakerke, Belgium) and XLSTAT for Windows XP (Addinsoft, Paris, France). Continuous variables were compared using an independent-sample analysis of variance (ANOVA) or Mann–Whitney test (skewed data). For categorical variables, Chi-squared or Fisher’s exact tests were used to assess significant differences in proportions. All reported *P-*values are two-sided and were considered statistically significant if <0.05.

For the logistic regression, some variables were grouped in different categories: (1) G6PD deficiency enzyme activity into two groups: severe deficient (severe G6PDd, Classes I and II) and mild deficient/normal (mild/normal G6PD, Classes III, IV and V); (2) sampling locations in three areas: western Cambodia (Anlong Veang, Trapeang Prasat, Angkor Ban, Chamkar Samraong, Suon Koma, Ou Chra, Ta Sanh, Pramaoy, Oral, Takvit, Kaoh Sla and Satv Pong), eastern Cambodia (Barkeo, Kaev Seima, Voeun Sai, Khsem) and central Cambodia (Tuol Kork, Phnom Dek, Sandan); and, (3) ages in three groups: <five years, five to 14 years and >15 years.

Variables with *P-*values <0.25 from the bivariate analyses were initially introduced into the model and removed following a backwards-stepwise selection procedure to leave only those variables those with a *P-*values <0.05 in the final model. Odds ratios (OR) and their 95% confidence intervals (CI) are reported for these significant explanatory variables.

## Results

From September 2010 to September 2012, 2,408 malaria-positive patients were recruited from western (n = 1,732, 71.9%), eastern (n = 414, 17.2%), and central (n = 262, 10.9%) Cambodia. The mean age was 26.7 years (SD: ± 12.0) with a range of two to 81 years, distributed as follows: (i) 0.5% < five years, (ii) 9.9% five to 14 years, and (iii) 89.6% >15 years. The male: female ratio was 3.9:1.

### Parasitological parameters

**Figure 2:**
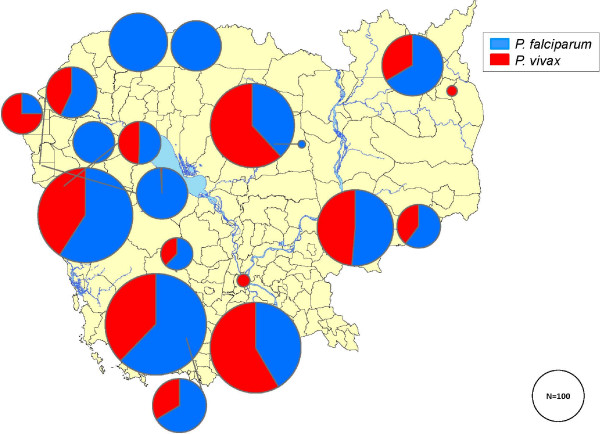
**Distribution of *Plasmodium falciparum *and *Plasmodium vivax *detected in 2,408 samples collected from malaria-infected individuals in 19 sites, Cambodia, 2010–2012.**

*Plasmodium falciparum* was present in 1,443 (59.9%) and *P. vivax* in 965 (40.1%) patients; species distribution is shown in Figure 2. Significant differences were found between *P. falciparum* and *P. vivax* infections for age (*P* <0.0001), sex (*P* <0.0001), G6PDd class (*P* <0.0001) and the detection haemoglobinopathy (Table 2).

**Table 2:** Patient characteristics by malaria species, Cambodia, 2010-2012

**Patients’ characteristics**		***Plasmodium vivax***	***Plasmodium falciparum***	***P*-value**
N		965	1443	-
Mean age in years (95% CI)		26.5 (25.8-27.2)	27.1 (26.3-27.7)	0.27
Age distribution (%)	<5 years	0.2	0.9	**<0.0001**
	5-14 years	9.2	11.9	
	>15 years	90.6	87.2	
Sex ratio (% female)		23.4	18.1	**<0.0001**
Mean G6PD activity in U/g Hb (95% CI)		11.86 (11.57-12.15)	11.45 (11.18-11.73)	0.05
G6PD Classes (%)	I (0% of normal)	0.10%	1.2%	**<0.0001**
	II (0.1-10% of normal)	4.9%	9.2%	
	III (10.1-60% of normal)	5.6%	6.0%	
	IV (60.1-150% of normal)	85.8%	77.7%	
	V (>150% of normal)	3.6%	6.0%	
Mean haemoglobin in g/dL (95% CI)		10.2 (10.0-10.4)	10.1 (9.9-10.2)	0.4
Mean white cell count in G/L (95% CI)		4.03 (3.86-4.20)	3.95 (3.81-4.09)	0.53
Haemoglobin electrophoresis profile (%)	Normal	56.1	61.6	**0.05**
	Hb E - homozygous	6.1	4.9	
	Hb E - heterozygous	33.5	30.1	
	Heterozygous E - α-thalassaemia	4.1	3.3	
	Heterozygous Hb E - β-thalassaemia	0.0	0.1	
	β-thalassaemia	0.0	0.1	

By logistic regression vivax malaria was: (i) more frequent in female patients (OR = 1.36, 95% CI: 1.07-1.72), but (ii) less frequent in severe G6PDd patients (OR = 0.27, 95% CI: 0.16-0.45) and patients with a haemoglobinopathy (OR = 0.74, 95% CI: 0.62-0.88).

### Haematological parameters

For all patients, the mean Hb concentration was 10.1 g/dL (95% CI: 9.9-10.2) and was significantly lower in: (i) younger age groups: 8.1 g/dL < five years, 8.7 g/dL five to 14 years, and 10.4 g/dL >15 years (*P* <0.001), (ii) females 9.1 g/dL *vs* males 10.5 g/dL (*P* <0.001), (iii) severe (Class I and II) G6PDd: 10.0 g/dL *vs* 10.7 g/dL for mild/normal G6PD, *P* = 0.004), (iv) patients with homozygous E: 8.8 g/dL *vs* 10.2 g/dL (normal profile) and *vs* 10.1 g/dL heterozygous HbE/heterozygous HbE and α-or β-thalassaemia (*P* <0.001). The mean Hb concentrations by G6PDd classes were: 11.1 g/dL for Class I (n = 18), 10.6 g/dL for Class II (n = 179), 9.5 g/dL for Class III (n = 140), 10.2 g/dL for Class IV (n = 1,949) and 8.0 g/dL for Class V (n = 122).

### G6PD activity

**Figure 3:**
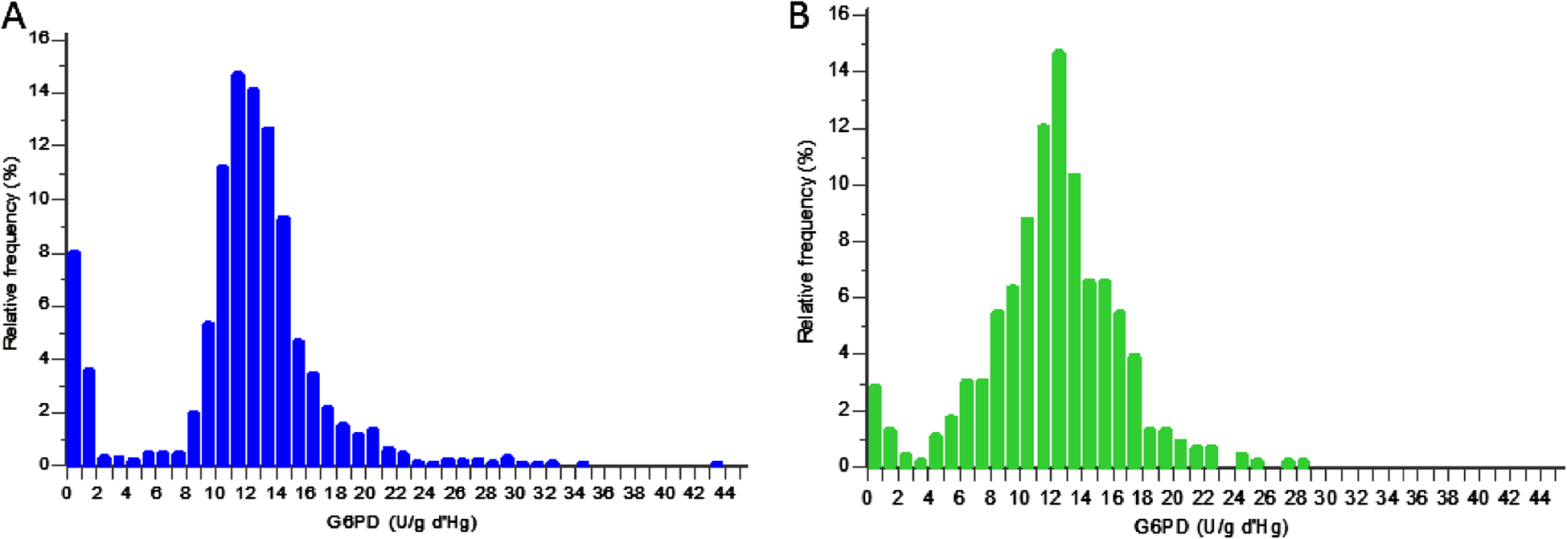
**Distribution of the G6PD enzymatic activity (U/g Hb) values of 2,408 samples collected from malaria-infected individuals in 23 sites, Cambodia, 2010–2012.** (Panel **A**: Male population and Panel **B**: Female population).

**Figure 4:**
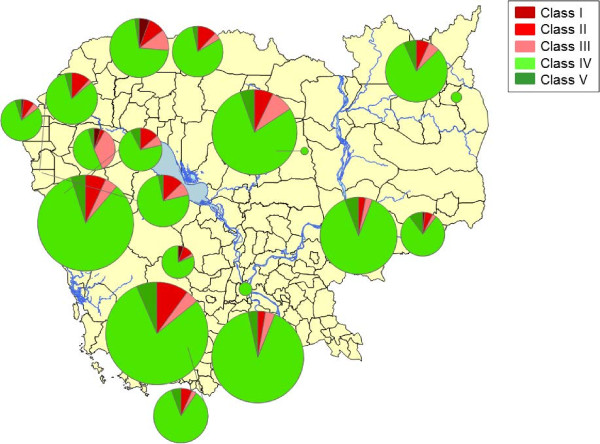
Distribution of the WHO G6PDd classes observed in 2,408 samples collected from malaria-infected individuals in 19 sites, Cambodia, 2010–2012.

**Figure 5:**
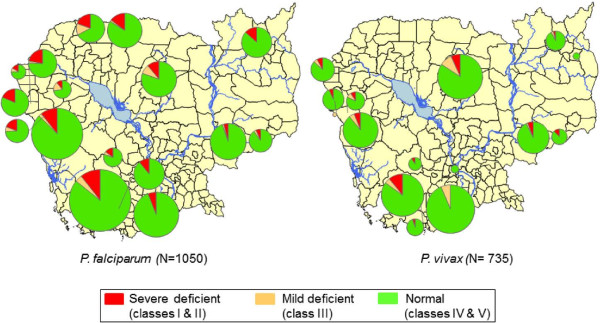
**Prevalence of G6PD severe deficiency (WHO classes I and II) observed in samples collected from malaria-infected males in 19 sites, according to *Plasmodium *species, Cambodia, 2010–2012.**

The mean G6PD enzyme activity was 11.6 (CI 95%: 11.4-11.8) U/g Hb (Figure 3); the proportions by WHO G6PD classes were: 0.7% (Class I), 7.4% (Class II), 5.8% (Class III), 80.9% (Class IV) and 5.1% (Class V). Class I and II G6PDd were more common in males: 21.4% *vs* 8.7% (*P* <0.0001). G6PDd was found more commonly in western compared to eastern Cambodia (Table 3 and Figures 4 and 5).

**Table 3:** Spatial distribution of severe deficiency (classes I and II) and severe/mild deficiency (classes I, II and III) in malaria-infected patients by provinces, Cambodia, 2010-2012

**Areas**	**Provinces**	**No.**	**Severe deficiency**			**Severe/Mild deficiency**		
			**No.**	**%**		**No.**	**%**	
Western	Oddar Manchay	212	29	13.7%	9.1%	47	22.1%	15.0%
	Battambang	187	20	10.7%		50	26.7%	
	Pailin	205	26	12.7%		37	18.0%	
	Pursat	323	24	7.4%		38	11.7%	
	Kampong Speu	37	6	16.2%		7	18.9%	
	Kampong Som	368	38	10.3%		53	14.4%	
	Kampot	400	15	3.7%		28	7.0%	
Central	Phnom Penh	8	0	0,0%	6.9%	0	0,0%	15.3%
	Preah Vihear	254	18	7.1%		40	15.7%	
Eastern	Rattanakiri	138	9	6.5%	5.1%	17	12.3%	8.9%
	Kratie	208	7	3.4%		13	6.2%	
	Mondolkiri	68	5	7.4%		7	10.3%	
**Cambodia**		**2,408**	**197**	**8.2%**		**290**	**14.0%**	

Logistic regression showed that Class I and II deficiency was less likely in (i) females (OR = 0.27, 95% CI: 0.23-0.67, *P* = 0.0007), and (ii) vivax-infected patients (OR = 0.45, 95% CI: 0.32-0.64, *P* <0.0001), but (iii) more likely in haemoglobinopathy patients: (OR = 1.5, 95% CI: 1.1-2.1, *P* = 0.0008).

### Haemoglobinopathies

The proportion of patients with abnormal haemoglobins was 41.4% (997/2,408). Heterozygous Hb E accounted for 862/997 (86.4%) of all the haemoglobinopathies. The prevalence of homozygous HbE (5.6%) was highest in MondulKiri province (13.2%), following by Preah Vihear province (9.8%), Battambang province (7.0%) and Sihanouk province (6.8%). The prevalence of heterozygous HbE (alone or with thalassaemia trait) (35.8%) ranges from 30.0% in Kampot province to 49.3% in Ratanakiri province.

## Discussion

For countries moving towards elimination of *P*. *vivax* and artemisinin-resistant *P*. *falciparum*, primaquine is an essential drug [3,29,30]. Primaquine is needed urgently in Cambodia but its safe introduction in the health system remains an important challenge due to the scarcity of G6PDd data, the lack of an inexpensive, reliable point-of-care test to detect G6PDd individuals [24] and the lack of data of any primaquine dose in malaria-infected patients. The recent WHO recommendation that G6PD testing may not be needed for low dose primaquine [8] is not yet supported by substantial evidence from Cambodia and other countries that have severe forms of G6PDd, nor are there yet any data from Cambodia on weekly primaquine dosing for G6PDd vivax-infected patients.

To date, most of G6PD studies in Cambodia have been conducted in a single site or in particular healthy populations (soldiers, children) using different methods to measure G6PD enzyme activity and different enzyme cut-offs to classify individuals as G6PDd (Table 1) [20,23,24,26,28], while the most useful G6PDd data should come from malaria-infected patients.

Health centres distributed throughout Cambodia were selected for geographical completeness in this study; although not a randomized sample, the species ratio was broadly similar to the recent national data. Most patients were young male adults (50% were aged 18–30 years), with anaemia (mean Hb about 10 g/dL and 74% had Hb <12 g/dL). Data observed in the present study show that anaemia was more frequent in children under five years of age, in females, in patients with G6PDd or with HbE-related haemoglobinopathies.

The high proportion of malaria-infected, anaemic patients reinforces the importance of not only detecting G6PDd but also to measure the haemoglobin concentration before starting primaquine because 0.75 mg/kg of primaquine, the recommended weekly dose in G6PDd vivax patients, has produced falls of 20-30% in haemoglobin concentration [21,31].

Before deploying primaquine in Cambodia, carefully conducted safety studies are needed for both low and weekly dose primaquine in falciparum and vivax patients, respectively, supported by studies to validate G6PD RDTs that have extremely low rates of missing G6PDd. Daily primaquine dosing in a RDT-diagnosed G6PD normal patient who is actually G6PDd would probably cause life threatening AHA in severe Southeast Asian G6PDd variants and may lead to a fall of confidence in primaquine by the population [32].

The G6PDd prevalence (13.9%) reported here is very similar to previous studies in healthy individuals [20,23,24,26-28] (Table 1): 8.1% for severe G6PDd and 5.8% for mild G6PDd and the mean sample G6PD enzyme activity of 11.6 U/g Hb is very close to the 11.8 U/g Hb reported previously by this laboratory in healthy individuals [24].

As expected, severe G6PDd was more frequent (~three-fold) in males whilst mild G6PDd was more frequent in females (~two-fold). G6PDd differs geographically with severe deficiency more common in western Cambodia. These data are of major concern for the safe use of primaquine as G6PDd is most prevalent in these areas, especially Pailin province, where artemisinin-resistant falciparum parasites are present and emerging, where *P*. *vivax* is still substantial, and where primaquine is urgently needed.

Haemoglobinopathies were also very common, affecting just under half of malaria patients and was associated with moderate anaemia. Heterozygous HbE was the most frequent haemoglobinopathy (86.4% of all haemoglobinopathies) and was more evenly distributed across Cambodia compared to G6PDd. Interestingly, like severe G6PDd, it protected patients against vivax malaria, consistent with data from others [33].

## Conclusions

This study has shown that G6PDd prevalence rates and enzyme activity in malaria patients are consistent with those in healthy individuals and that severe G6PDd is prevalent mostly in western areas of Cambodia where artemisinin resistance is present. Haemoglobinopathies were common and contributed to anaemia but also to protection against *P. vivax*. Many malaria patients were anaemic, raising questions whether primaquine-induced AHA would be well tolerated. Combined primaquine safety and G6PD point of care evaluation studies are needed most urgently in Cambodia.

## Competing interests

The authors declare that they have no competing interests.

## Authors’ contributions

DM contributed to the design and coordination of the study, assisted with data entry, analysis and interpretation. SKi supervised the field study. NK, CB, PC, LC, BW, AK and PT were involved in laboratory work. NK, CB, WRJT and DM wrote the first draft of the paper. SKh, SS, RL, SL, SM, NC, CMC and SD gave constructive advice. All authors read and approved the final manuscript.

## Authors’ information

Walter RJ Taylor and Didier Ménard share senior authorship.
